# Supplementary material for: Multilayer Network Analysis of Nuclear Reactions
Source: Sci Rep. 2016 Aug 25;6:31882. doi: 10.1038/srep31882 (PMC4997254; doi:10.1038/srep31882)
Supplement: Supplementary Information [file srep31882-s1.pdf]

Supplementary Information

# Multilayer Network Analysis of Nuclear Reactions

**Liang Zhu[1,2], Yu-Gang Ma[1,3,*], Qu Chen[4,5], and Ding-Ding Han[4,5]**

[1]Shanghai Institute of Applied Physics, Chinese Academy of Sciences, Shanghai 201800, China

[2]University of Chinese Academy of Sciences, Beijing 100049, China

[3]ShanghaiTech University, Shanghai 200031, China

[4]School of Information Science and Technology, East China Normal University, Shanghai 200241, China

[5]Shanghai Key Laboratory of Multidimensional Information Processing, East China Normal University, Shanghai 200241, China

*ygma@sinap.ac.cn

# Supplementary Figure 1

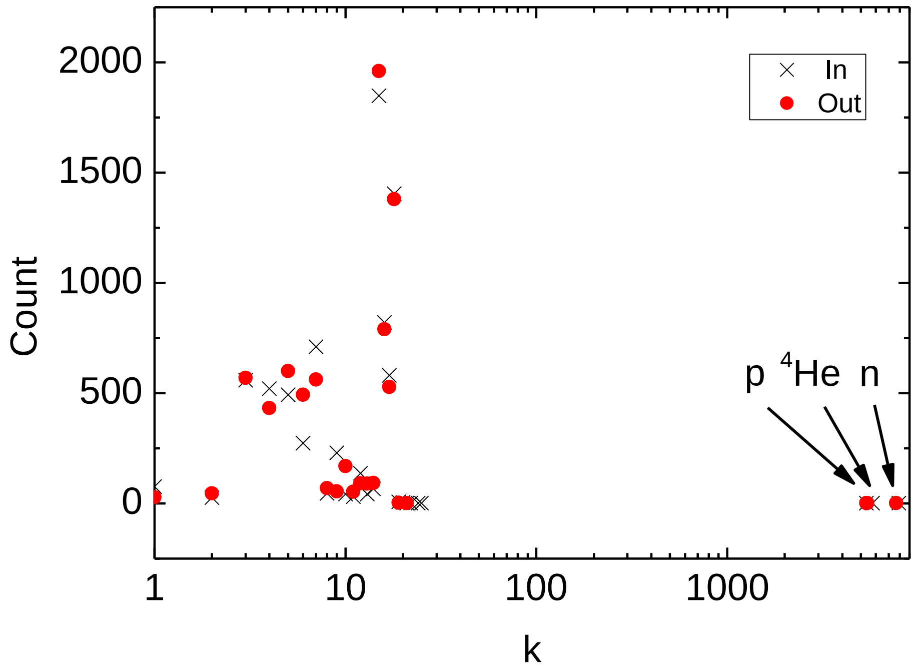


**The in- and out-degree distributions of the nuclear reaction network, with n, p and $^4He$ treated as normal nodes.** The in- and out-degree of these 3 particles are all greater than 5000. The degree distribution is highly biased, making the network analysis difficult. The number of reactions that consumes n, p or $^4He$ is 48515 (out of 82851), so it is reasonable to treat them as currency particles instead.

# Supplementary Figure 2

**a**

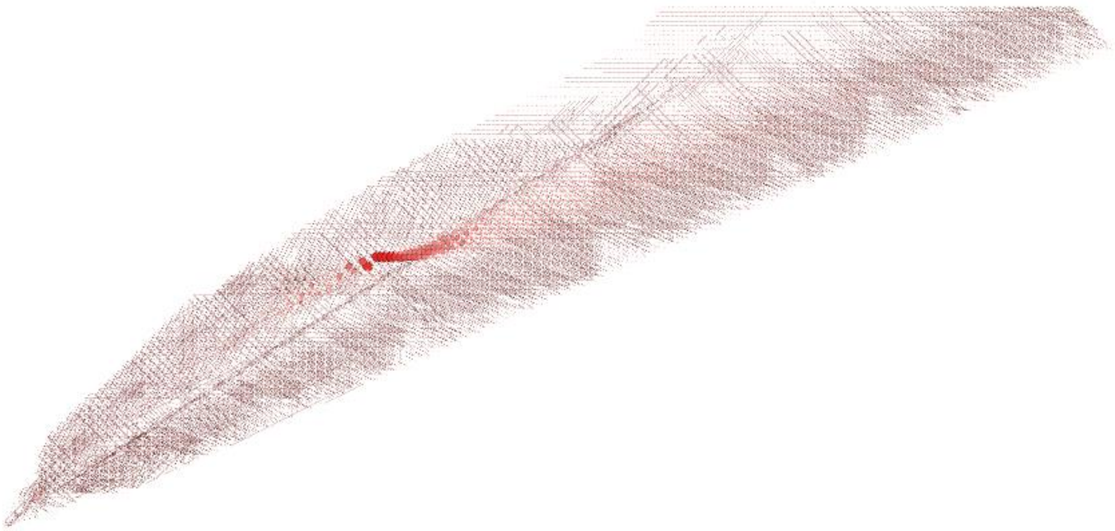

**b**

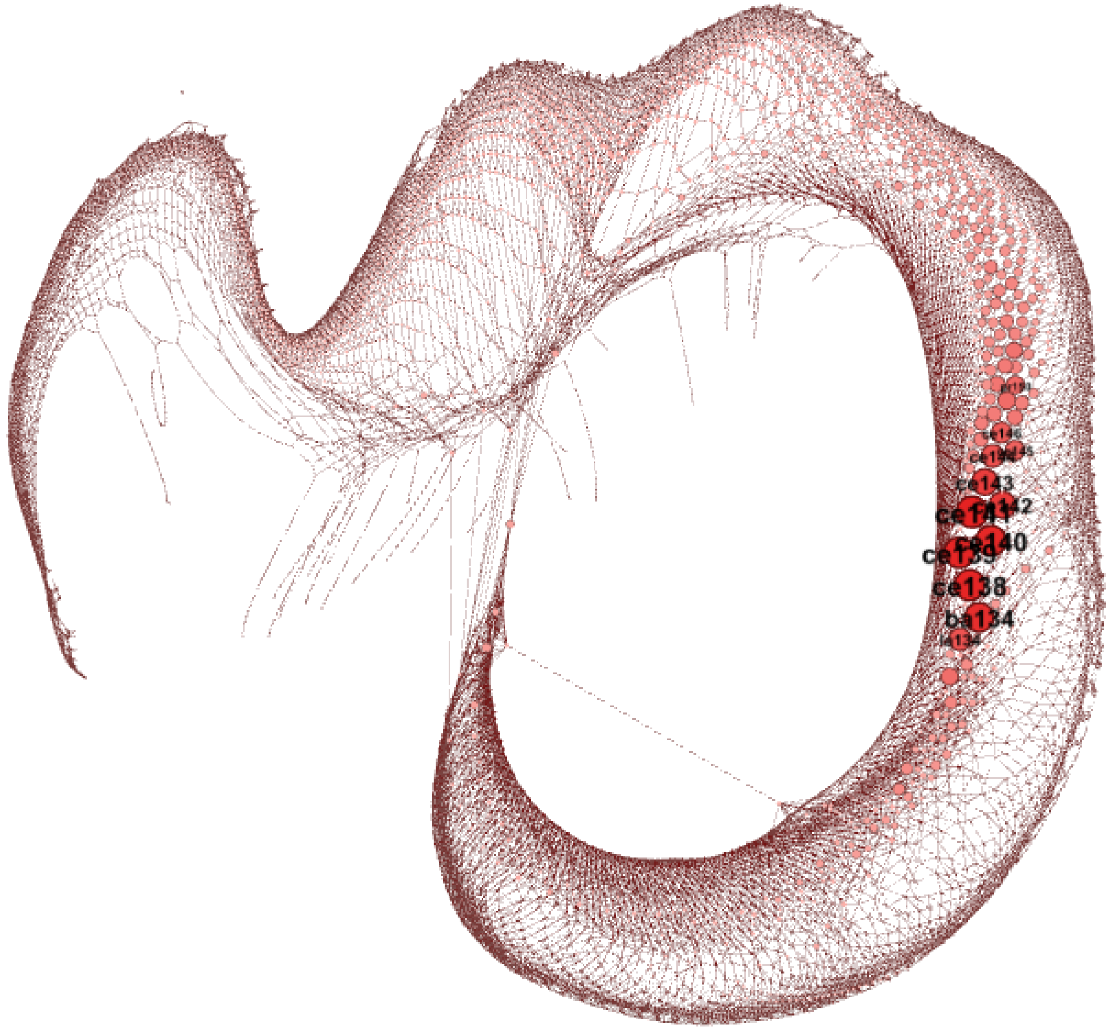

**The topology of the aggregated nuclear reaction network plotted with Gephi.** (a) geo layout with x-axis being N and y-axis being Z, (b) ForceAtlas layout. The nodes with larger sizes have higher betweeness centrality. If we define link length by its spatial distance in the Z-N plane, we would have only a tiny number of long range links, like $^{114}$Ba, $^{221}$Ra, $^{233}$U, $^{235}$U connecting lighter areas through some C, N ,O nuclides.

# Supplementary Figure 3

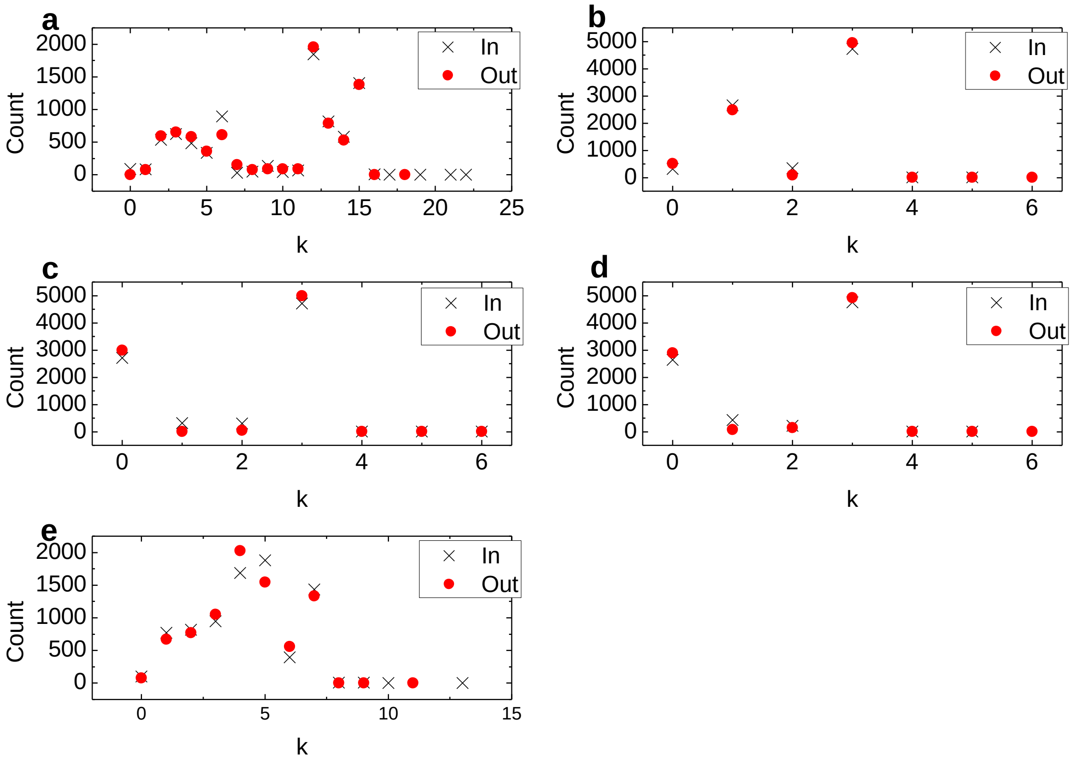


**The degree distribution of the nuclear reaction network with n, p and $^{4}$He treated as currency particles.** (a) aggregated network, (b) n-layer, (c) p-layer, (d) h-layer and (e) r-layer. Unlike other networks in the empirical studies, the degree of the nuclear reaction network has a narrow distribution with a peak around k=12, and does not show power-law characteristics. The correlation between in- and out-degree is high. There is a peak at k=3 in n-, p- and h-layer, and these 3 layers all have similar distributions and are much narrower than that of the aggregated network. While in r-layer, the distribution is distinct from the other 3 layers.

# Supplementary Figure 4

**a**

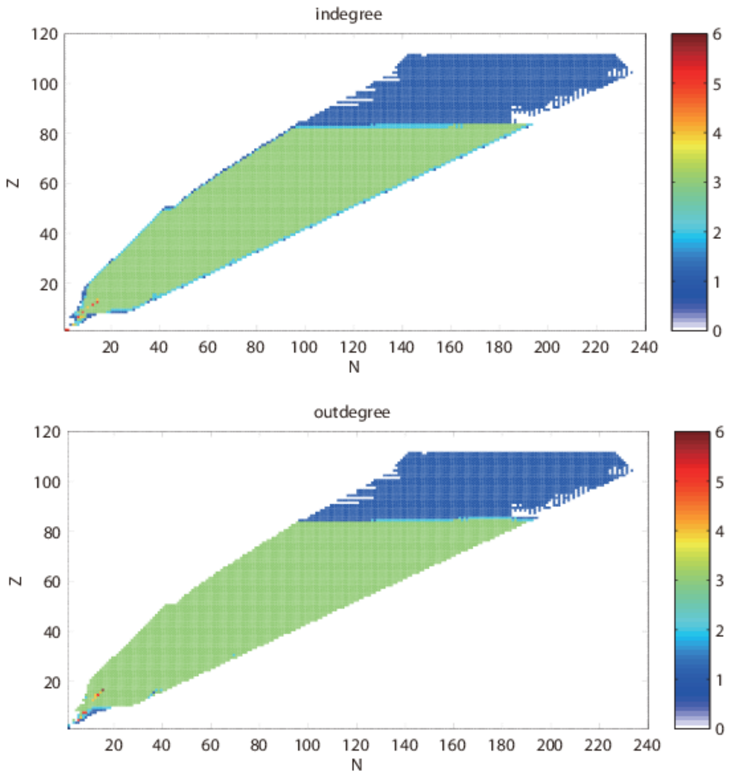


**b**

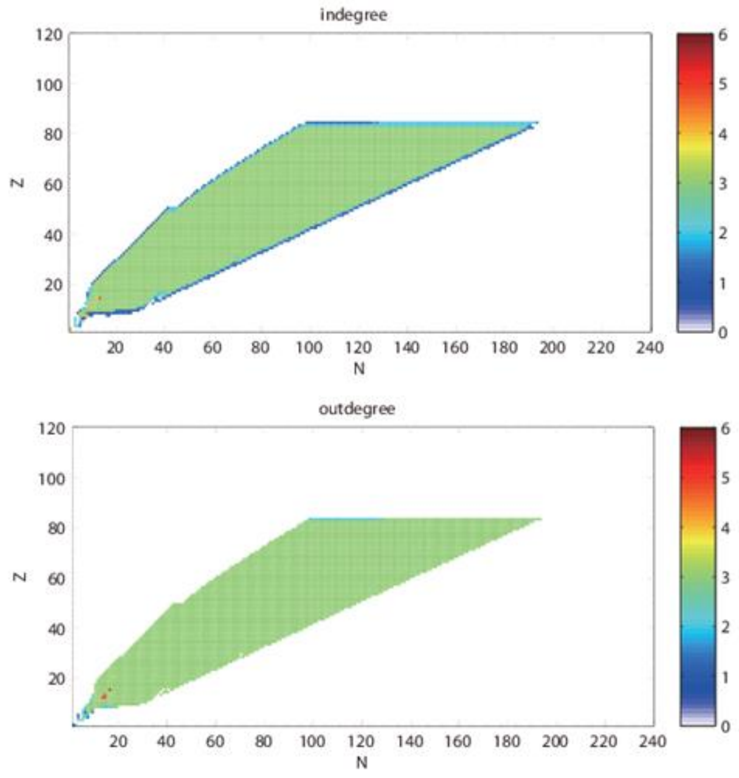

**c**

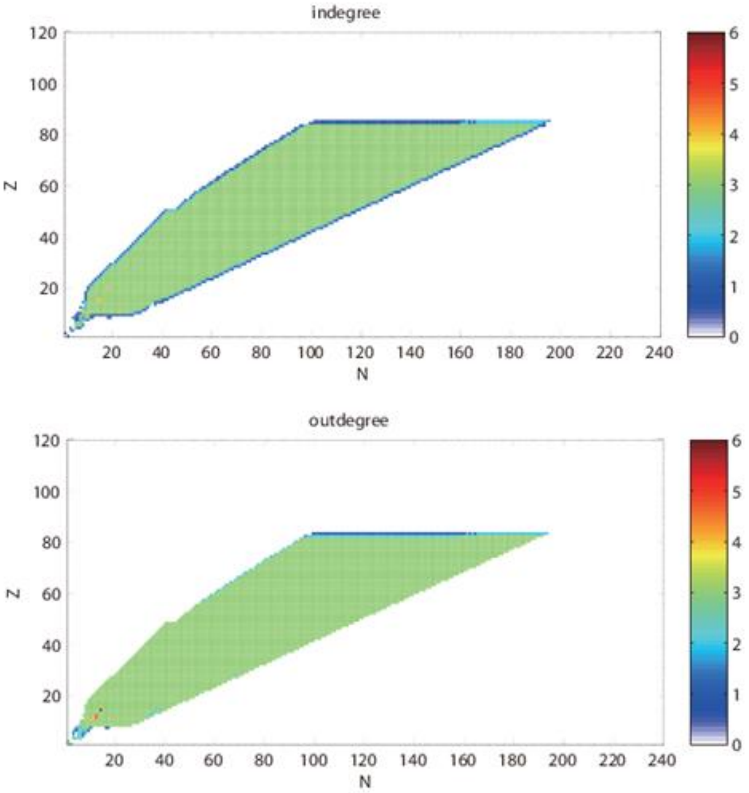


**Degree distributions of (a) n-layer, (b) p-layer and (c) h-layer on a Z-N plane.** The in-degree and out-degree are calculated for each nuclide and the values are indicated by color, with x-axis being the number of neutrons (N) in that nuclide and y-axis being protons (Z).

# Supplementary Figure 5

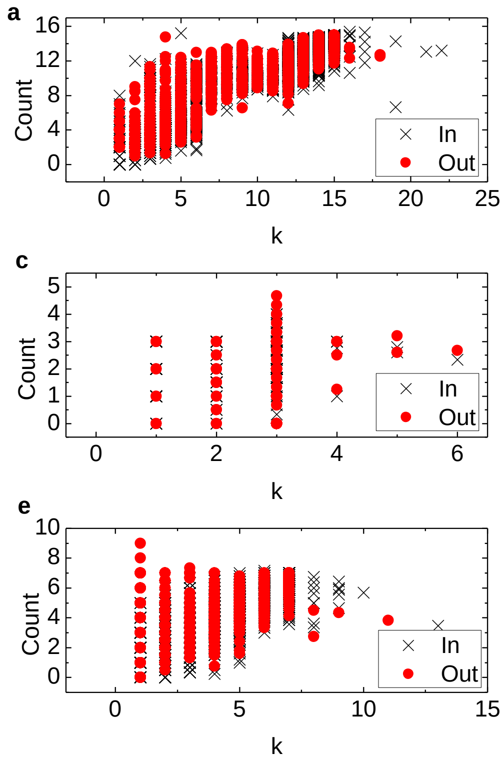


**The $k_{nn}$-k correlation of the nuclear reaction network.** (a) aggregated network, (b) n-layer, (c) p-layer, (d) h-layer and (e) r-layer. The aggregated network shows positive correlation between $k_{nn}$ and k. The r-layer has correlation to a certain extent, while the other 3 layers has little.

# Supplementary Figure 6

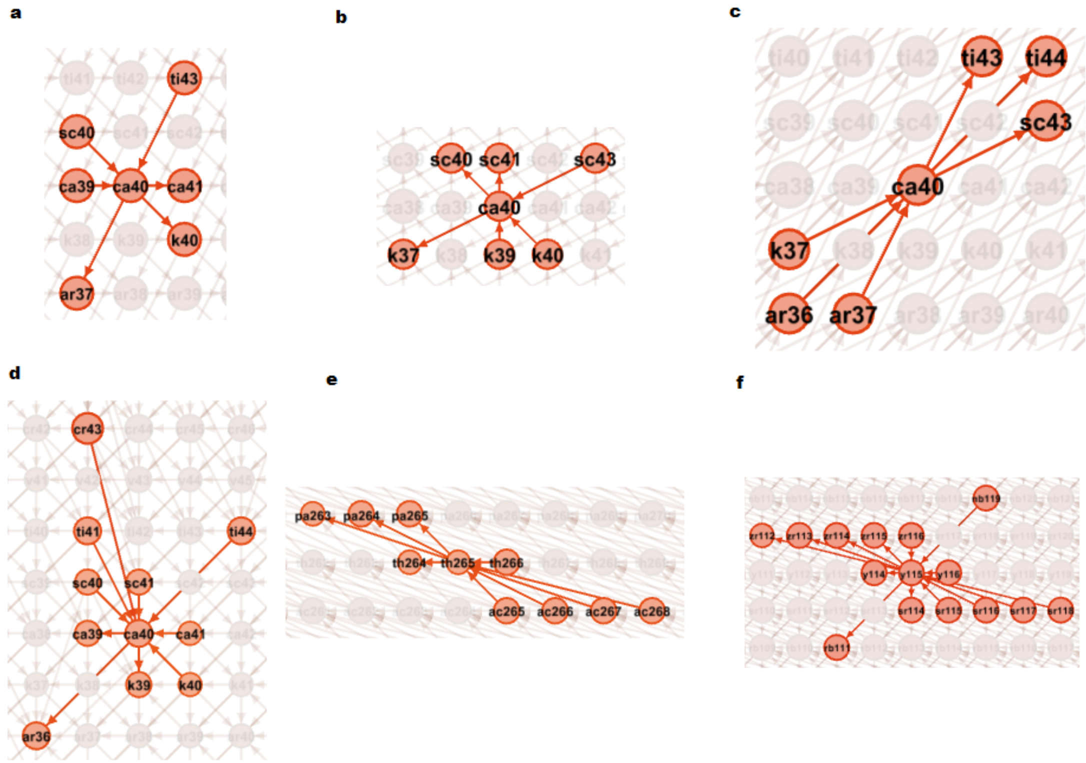


**Typical motifs of each layer.** (a) n-layer, (b) p-layer, (c) h-layer and r-layer have mainly 3 kinds of motifs (d), (e), (f).

# Supplementary Figure 7

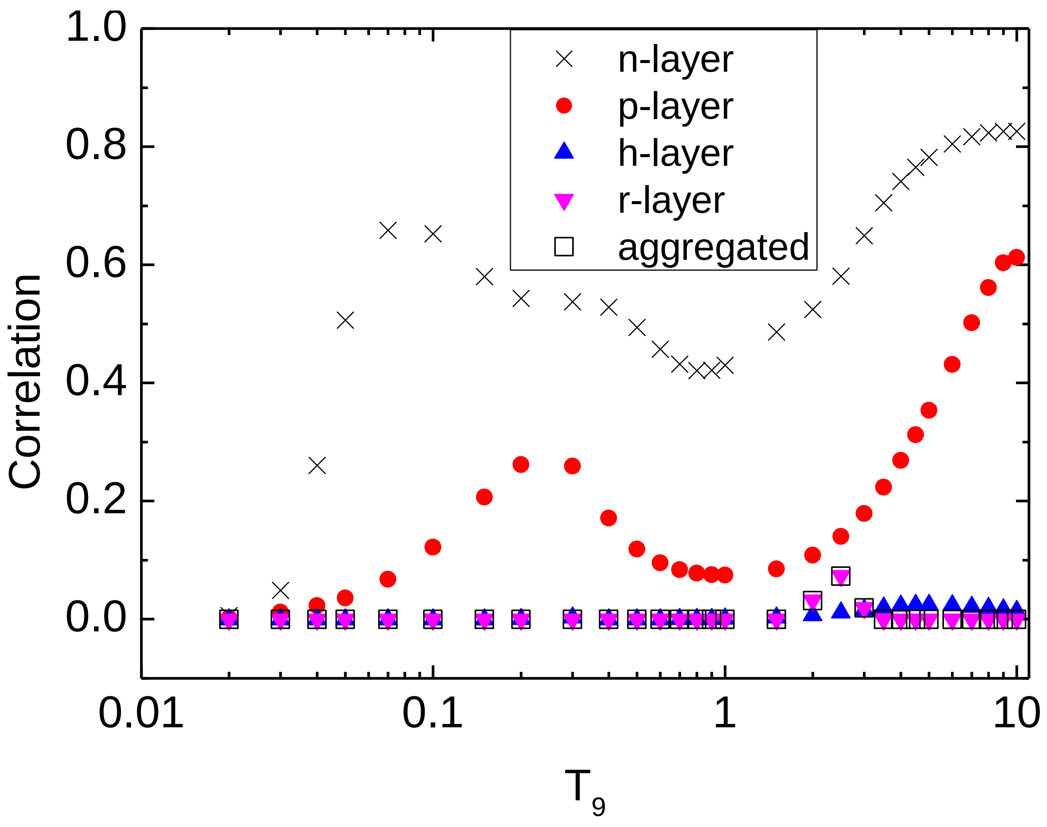


**The correlation between in- and out-strength as a function of temperature.** For n- and p-layer, where neutron and proton capture process take place, the correlation varies extensively, which could be the source of complexity. Around $T_9$=3, the correlation in r-layer has a peak, but the connection between out-strength and half-life is still unique. The overall correlation of the network is low.

# Supplementary Figure 8

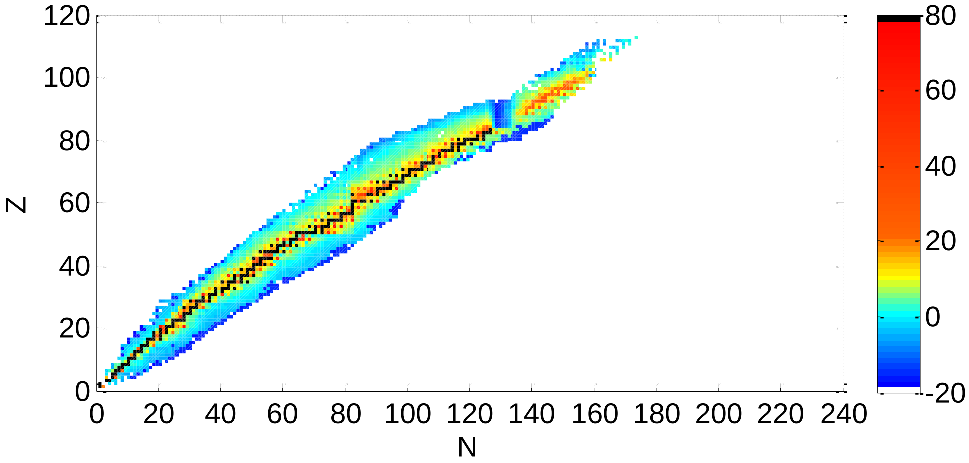


**The half-life data from Nudat in a Z-N panel in compare with panel (b) of Fig. 6.** $\ln(t^h)$ of a nuclide is indicated by color, with x-axis being the number of neutrons (N) in that nuclide and y-axis being protons (Z).

# Supplementary Note 1

The dataset specification can be found at JINA REACLIB website (http://groups.nscl.msu.edu/jina/reaclib/db/). The reactions are classified into 11 kinds (chapters) according to the number of reactants and products, and the number of each kind of reaction is counted as in the table below. The reactions are classified as Chapter 1, 2, 4 and 5, which only have one or two reactants and one or two products at the same time, covering 93.53% of the whole dataset.

| Chapter | Equation | Count | Percentage |
|---|---|---|---|
| **1** | e1 → e2 | 6276 | 7.58% |
| **2** | e1 → e2 + e3 | 22747 | 27.46% |
| **3** | e1 → e2 + e3 + e4 | 3112 | 3.76% |
| **4** | e1 + e2 → e3 | 18075 | 21.82% |
| **5** | e1 + e2 → e3 + e4 | 30378 | 36.67% |
| **6** | e1 + e2 → e3 + e4 + e5 | 24 | 0.03% |
| **7** | e1 + e2 → e3 + e4 + e5 + e6 | 6 | 0.01% |
| **8** | e1 + e2 + e3 → e4 | 7 | 0.01% |
| **9** | e1 + e2 + e3 → e4 + e5 | 24 | 0.03% |
| **10** | e1 + e2 + e3 + e4 → e5 + e6 | 6 | 0.01% |
| **11** | e1 → e2 + e3 + e4 + e5 | 2196 | 2.65% |

# Supplementary Note 2

The average degree of the aggregated nuclear network is to 19.233. The average shortest path length is 41.625 comparing to the size 8048. The clustering coefficient is 0.264. The network is a dense one without ‘small-world’ characteristics and is not structured like other systems usually studied. The topologies shown in Supplementary Figure 2 and the corresponding degree distributions in Supplementary Figure 3 and 4 demonstrate that the n-, p- and h-layer are quite regular as binary networks. The $k_{nn}$-k correlation in Supplementary Figure 5 shows assotativity of the aggregated network, but the reaction rate has a broad distribution and the in- and out-strength correlation of nodes vary with temperature in Supplementary Figure 7, making the network uncommon again. Note that the correlation is calculated with Pearson correlation coefficient.
